# Dexmedetomidine Directs T Helper Cells toward Th1 Cell Differentiation via the STAT1-T-Bet Pathway

**DOI:** 10.1155/2021/3725316

**Published:** 2021-08-09

**Authors:** Daoyun Lei, Li Liu, Songhui Xie, Haiyan Ji, Yanxing Guo, Tieliang Ma, Chao Han

**Affiliations:** ^1^Department of Anesthesiology, The Affiliated Yixing Hospital of Jiangsu University, Yixing, Jiangsu, China; ^2^Department of Anesthesiology, Zhongda Hospital Southeast University, Nanjing, Jiangsu, China; ^3^Yixing Clinical College, Medical College of Yangzhou University, Yixing, Jiangsu, China

## Abstract

Dexmedetomidine is an *α*2 adrenergic receptor agonist that has been reported to modulate the polarization of CD4+ T cells. However, the underlying mechanisms by which dexmedetomidine induces T-helper 1 (Th1) cell differentiation remain poorly understood. The aim of this study was to explore the potential mechanisms through which dexmedetomidine can induce Th1 cell differentiation. Purified CD4+ T cells were stimulated with anti-CD3/anti-CD28 and then treated with dexmedetomidine. Flow cytometry analysis was adopted to measure the concentration of Th1 cells. Enzyme-linked immunosorbent assay (ELISA) and real-time quantitative polymerase chain reaction (qPCR) were performed to detect protein levels and mRNA expression, respectively, of IFN-*γ* and IL-4. Western blotting was used to determine the phosphorylation of signal transducer and activator of transcription 1 (STAT1) and T-bet expression. The Th1 cell subset and IFN-*γ* levels were elevated in the dexmedetomidine-induced CD4+ T cells. Dexmedetomidine enhanced the phosphorylation of STAT1 and the expression of T-bet in the CD4+ T cells. Atipamezole (an *α*2 adrenergic antagonist) and fludarabine (a STAT1 inhibitor) reversed the dexmedetomidine-induced Th1 cell differentiation. These results suggested that dexmedetomidine induced Th1 cell differentiation via the STAT1-T-bet signaling pathway.

## 1. Introduction

Surgical trauma and tumor microenvironments can suppress the innate immunity of patients, as well as increase the postoperative infection rate and tumor metastasis by inducing CD4+ T cells toward a T-helper 2 (Th2) cell fate [1, 2]. Conversely, regulating Th1 cell fate enhances the function of natural killer cells to inhibit the growth of breast cancer cells [3] and relieves the symptoms of infection [4]. As a selective *α*2 receptor agonist, dexmedetomidine is widely used in surgery and anesthesia for its complex pharmacological effects, such as sedation, analgesia, and suppression of anxiety and sympathetic tone [5]. Clinical studies [6, 7] found that dexmedetomidine increases serum IFN-*γ*, suggesting enhancement of Th1 cell fate in surgery patients. In addition, dexmedetomidine has the same effect in rat spinal cord injury [8]. Dexmedetomidine may regulate Th1 cell differentiation to relieve the immunosuppressant effect induced by surgical trauma, but the underlying mechanism remains unclear.

After being stimulated by antigens and cytokines, CD4+ T cells acquire the ability to differentiate into different cell subtypes with specific effects. These cell subtypes adjust the immune system to exert corresponding immune effects according to different pathogens [9]. Th1 cells secrete mainly IL-2, IFN-*γ*, and TNF-*α*, regulating the cell-mediated immune response [10, 11]. Th2 cells characteristically secrete IL-4, IL-5, and IL-10, upregulate the function of B cells, and participate in humoral immune responses. T helper cells with differentiated ability selectively polarize the expression of certain T helper cell-related genes. Positive feedback enhances their characteristic immune function and inhibits the differentiation of other cell subtypes. Failure to form an appropriate polarization reaction could increase the infection rate and induce autoimmune or allergic diseases [10].

T-bet is the most critical transcription factor in the regulation of Th1 cell differentiation. Many transcription factors, such as NFAT, AP-1, STAT4, and NF-*κ*B, participate in Th1 cell differentiation [12, 13]. However, their functions are dependent on T-bet constructing an epigenetic environment for Th1 characteristic genes [14]. T-bet binds directly to the DNA sequence in the IFN-*γ* promoter to enhance the expression of IFN-*γ* and positively increase the expression of T-bet after stimulation by the TCR signal. This phenomenon promotes Th1 cell differentiation. Moreover, T-bet downregulates Th2 cell differentiation by inhibiting GATA3 binding to the characteristic genes [15]. Enhancing T-bet reverses T-bet deficiency-inhibited Th1 cell differentiation [16] and weakens the differentiation of Th2 cells, as well as ectopic expression of T-bet [17].

STAT1, a member of the signal transduction and transcription activator (STAT) family, is usually inactive in the cytoplasm [18]. Once phosphorylation occurs, STAT1 can positively enhance Th1 cell differentiation following translocation from the cytoplasm to the nucleus, where it induces specific expression by binding to IFN-*γ* [19]. Inhibiting STAT1 activity by downregulating STAT1 phosphorylation levels significantly reduces the concentration of Th1 cells compared to Th2 cells [19, 20]. The content of Th1 cells and the expression of IFN-*γ* or T-bet were found to be significantly reduced in STAT1^−/−^ mice [21]. Meanwhile, inhibiting the phosphorylation level of STAT1 inhibited T-bet expression [22], suggesting that STAT1 phosphorylation may be the key node in T-bet expression.

The aims of this study were to determine the direct effect of dexmedetomidine on inducing Th1 cell differentiation and reveal the underlying mechanism.

## 2. Methods and Material

### 2.1. Mice

C57BL/6J mice were obtained from the Animal Care Committee of Nanjing Medical University. All mice were housed in specific pathogen-free conditions according to the Animal Care Committee of Nanjing Medical University. Only male mice were used experimentally at 6–8 weeks of age. All animal procedures were approved by the Institutional Animal Care and Use Committee of Nanjing Medical University. The study was conducted in accordance with the Basic and Clinical Pharmacology and Toxicology policy for experimental and clinical studies.

### 2.2. Cell Purification and Culture

Mice were killed by cervical dislocation under sterile conditions. The spleens were obtained from the mice by opening the abdominal cavity. The spleens were gently ground into a single cell suspension. To obtain purified CD4+ T cells, CD4 (L3T4) microbeads (MiltenyiBiotec, Germany) were used according to the manufacturer's protocol to obtain CD4+ T cells with a purity > 90%. Purified CD4+ T cells (1 × 10^6^ cells) were activated with 3 *μ*g/plate-bound anti-CD3 (BD Biosciences, USA) and 5 *μ*g/soluble anti-CD28 (BD Biosciences, USA) for 3 days. Different concentrations of dexmedetomidine (0, 0.01, 0.1, 1, and 10 nM) were added to stimulate the T cells for another 24 hours. To further explore the underlying mechanism, atipamezole (1 nM, CSNpharm, China) or fludarabine (100 nM, CSNpharm, China) was added for 24 hours to stimulate the T cells before using dexmedetomidine.

### 2.3. Flow Cytometry

Following treatment of purified CD4+ T cells with different drugs, the concentrations of Th1 cells and Th2 cells were investigated by flow cytometry (BD Bioscience, USA). To facilitate the intracellular staining of T helper cells, all cells were stimulated with Cell Activation Cocktail (with Brefeldin A) (BioLegend, USA) for 5 hours before detection. Staining of T helper cells for flow cytometry requires two steps: extracellular staining and intracellular staining. After the harvested cells were washed with FACS buffer, FITC-conjugated anti-mouse CD4 (BioLegend, USA) was added to the cells for 30 minutes at RT. To decrease the nonspecific Fc receptor-mediated Ab staining, cells were preincubated with mouse IgG for 20 minutes at 4°C. Then, the FIX&PERM Kit (FMS, China) was used according to the manufacturer's instructions to fix and permeabilize the cells. APC-conjugated anti-mouse IFN-*γ* (BioLegend, USA) and PE-conjugated anti-mouse IL-4 (BioLegend, USA) were added to bind to intracellular cytokines. Th1 cells and Th2 cells were labeled with CD4 + IFN-*γ* and CD4 + IL-4, respectively.

### 2.4. Cytokine Protein Assay

After stimulation of the CD4+ T cells was completed, the supernatant was collected to detect the concentration of IFN-*γ* and IL-4 by ELISA according to the manufacturer's protocol (mouse IL-4 IFN-*γ* kit, MultiSciences, China; mouse IL-4 ELISA kit, MultiSciences, China). A standard plate reader was used to read absorbance at 450 nm. The establishment of the standard curve was based on the optical density (OD) measurements. The required cytokine concentrations were extrapolated from this standard curve.

### 2.5. RT and Real-Time PCR Analysis

To analyze the expression of cytokines, total RNA was extracted from CD4+ T cells using 1 ml TRIzol (Takara Bio, Shiga, Japan) reagent following the protocols supplied by the manufacturer. According to the manufacturer's instructions, total RNA was reverse transcribed into cDNA using PrimeScript RT Master Mix (Takara Bio). Then, SYBR® Green was used to conduct real-time PCR.

The following primer sets were used [23]: GAPDH: 5′-TGCAGTGGCAAA GTGGAGATT-3′ (forward) and 5′-TCGCTCCTGGAAGATGGTGAT-3′ (reverse); IFN-*γ*: 5′-GCAACAGCAAGGCGAAAAAG-3′ (forward) and 5′-TTCCTGAGGCTGGATTCGG-3′ (reverse); T-bet, 5′-CCATTCCTGTCCTTCACCG-3′ (forward) and 5′-CTGCCTTCTGCCTTTCCAC-3′ (reverse); GATA-3, 5′-GCCTGCGGACTCTACCATAA-3′ (forward) and 5′-AGGATGTCCCTGCTCTCCTT-3′ (reverse). GAPDH was used as the housekeeping gene for normalization, and target mRNA levels were quantified using the 2^–*ΔΔ*Ct^ method.

### 2.6. Western Blot

The stimulated cells were lysed on ice with RIPA buffer plus 1% phosphatase inhibitor and 1% protease inhibitor for 20 minutes. The cells were mixed with loading buffer and boiled for 8 minutes at 100°C. To detect protein expression, 10 *μ*L of samples was separated by 10% SDS-PAGE and transferred to the PVDF membrane. The membranes were blocked with 5% skimmed milk at room temperature for 2 hours. Then, the membranes were incubated with the primary antibodies at 4°C overnight and washed three times with PBST for 10 minutes. The following antibodies were used: anti-*β*-actin rabbit pAb (1 : 1,000, Cell Signaling Technology, USA), anti-STAT1 rabbit pAb (1 : 1000, HuaBio, China), and anti-p-STAT1 rabbit pAb (1 : 1,000, HuaBio, China). The membranes were incubated with HRP-goat anti-rabbit IgG (1 : 5,000, Abcam, USA) antibody for 1 hour at room temperature. The reaction complexes were visualized using enhanced chemiluminescence enhancement reagents.

### 2.7. Statistical Analysis

Statistical analysis was performed using GraphPad Prism 5 software. The data are shown as the mean ± standard error of the mean (SEM). Means were compared using Student's *t*-test for two groups or one-way ANOVA for multiple groups. *p* < 0.05 was considered significant.

## 3. Results

### 3.1. Dexmedetomidine Induces Th1 Cell Differentiation in Purified CD4+ T Cells

**Figure 1:**
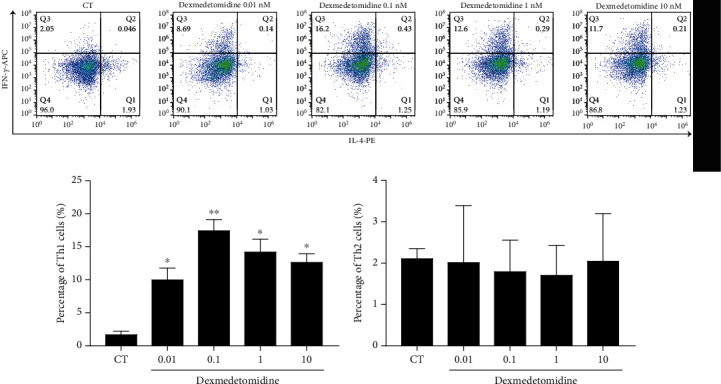
Dexmedetomidine induces Th1 cell differentiation in CD4+ T cells. CD4+ T cells were incubated with anti-CD3/CD28 and rIL-2 for 3 days. Then, different concentrations of dexmedetomidine (0, 0.01, 0.1, 1, and 10 nM) were added to stimulate CD4+ T cells for another 24 hours. Th1 and Th2 cell concentrations were measured (a) and analyzed (b) as described in Methods and Material. The data are representative of at least three independent experiments with consistent results. Student's *t*-test was performed to detect between-group differences. The results shown are the mean ± S.D. ^∗^*p* < 0.05, ^∗∗^*p* < 0.01.

The polarization reaction of activated CD4+ T cells can affect the immune status [10]. To determine whether activation of the *α*2 receptors on the surface of T cells regulates the differentiation of Th1 and Th2 cells, different concentrations of the *α*2 receptor agonist dexmedetomidine were administered to stimulate CD4+ T cells for 24 hours in vitro. Before adding dexmedetomidine, CD4+ T cells were activated by culturing them with CD3/CD28 antibodies and rIL-2 for 3 days. After all stimulations were complete, the flow cytometer was operated to monitor the differentiation of Th1 and Th2 cells. As shown in Figure 1, dexmedetomidine increased the concentration of Th1 cells in the CD4+ T cells, especially at a concentration of 0.1 nM, while it had no effect on the concentration of Th2 cells. These results suggest that dexmedetomidine regulates the polarization of CD4+ T cells, inducing Th1 differentiation but not Th2 differentiation.

### 3.2. Dexmedetomidine Enhances the Expression of the Th1 Characteristic Cytokine IFN-*γ* in Activated CD4+ T Cells

**Figure 2:**
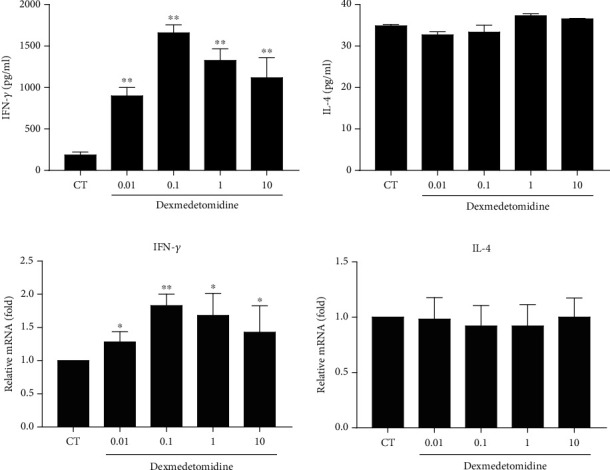
Dexmedetomidine enhances IFN-*γ* production by CD4+ T cells. After CD4+ T cells were incubated with anti-CD3/CD28 and rIL-2 for 3 days, different concentrations of dexmedetomidine (0, 0.01, 0.1, 1, and 10 nM) were administered for another 24 hours. IFN-*γ* and IL-4 production (a) were measured by ELISA. IFN-*γ* and IL-4 (b) mRNA expression was tested by real-time PCR as described in Methods and Materials. The data are representative of at least three independent experiments with consistent results. Student's *t*-test was performed to detect between-group differences. The results shown are the mean ± S.D. ^∗^*p* < 0.05, ^∗∗^*p* < 0.01.

Based on the knowledge that dexmedetomidine induces Th1 cell differentiation, we wished to determine whether dexmedetomidine would also regulate the expression of characteristic cytokines. ELISA and RT-PCR were adopted to measure the protein concentration and expression, respectively, of IFN-*γ* and IL-4. Dexmedetomidine enhanced the concentration and expression of IFN-*γ* but had no regulatory effect on IL-4 concentration or expression in activated CD4+ T cells (Figures 2(a) and 2(b)). These results indicated that dexmedetomidine not only induced Th1 cell differentiation but also enhanced the function of Th1 cells.

### 3.3. Dexmedetomidine Upregulates STAT1 Phosphorylation and T-Bet Expression in Stimulated CD4+ T Cells

**Figure 3:**
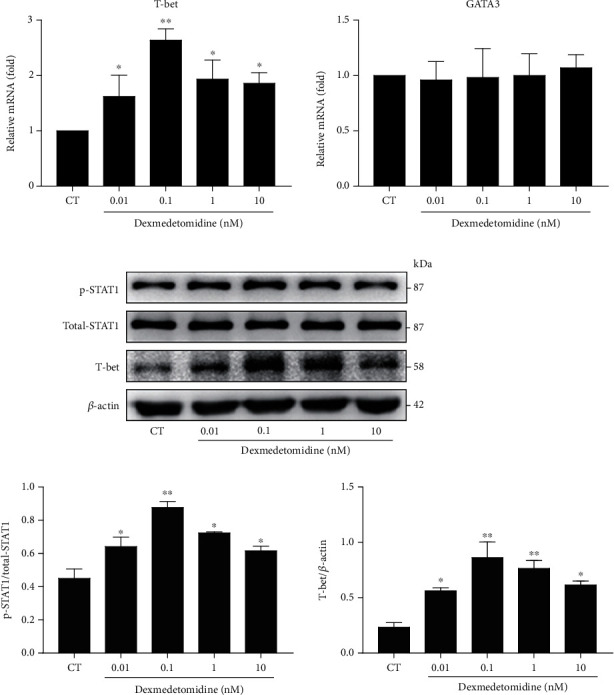
Dexmedetomidine results in increased STAT1 phosphorylation and T-bet expression. CD4+ T cells received anti-CD3/CD28 and rIL-2 treatment for 3 days. Different concentrations of dexmedetomidine (0, 0.01, 0.1, 1, and 10 nM) were added to induce genes and protein expression. (a) T-bet and GATA3 mRNA expressions were determined by real-time PCR. (b) STAT1 phosphorylation and T-bet expression were defined using Western blot. The data are representative of at least three independent experiments with consistent results. Student's *t*-test was performed to detect between-group differences. The results shown are the mean ± S.D. ^∗^*p* < 0.05, ^∗∗^*p* < 0.01.

Activated STAT1 enhances the expression of IFN-*γ* through phosphorylation to induce the differentiation of Th1 cells [19]. We speculated that dexmedetomidine would induce the differentiation of Th1 cells through activation of STAT1 phosphorylation. To reveal the potential mechanism of dexmedetomidine-induced Th1 cell differentiation, the level of STAT1 phosphorylation was tested by western blots. With different concentrations of dexmedetomidine to stimulate CD4+ T cells, the phosphorylation level of STAT1 was increased (Figure 3(b)). The expression of transcription factors is a key factor in T cell differentiation. T-bet and GATA3, as characteristic transcription factors in Th1 and Th2 cell differentiation, were detected by RT-PCR to determine expression at the mRNA level. Stimulation by dexmedetomidine significantly promoted the expression of T-bet without affecting the expression of GATA3 in CD4+ T cells (Figure 3(a)). The protein expression of T-bet was also monitored by western blots to clarify further that dexmedetomidine stimulation raised T-bet protein levels (Figure 3(b)). These findings showed that dexmedetomidine-induced Th1 cell differentiation may be mediated by enhancing STAT1–T-bet signaling.

### 3.4. Atipamezole Attenuates Dexmedetomidine-Induced Th1 Cell Differentiation and Cytokine Production

**Figure 4:**
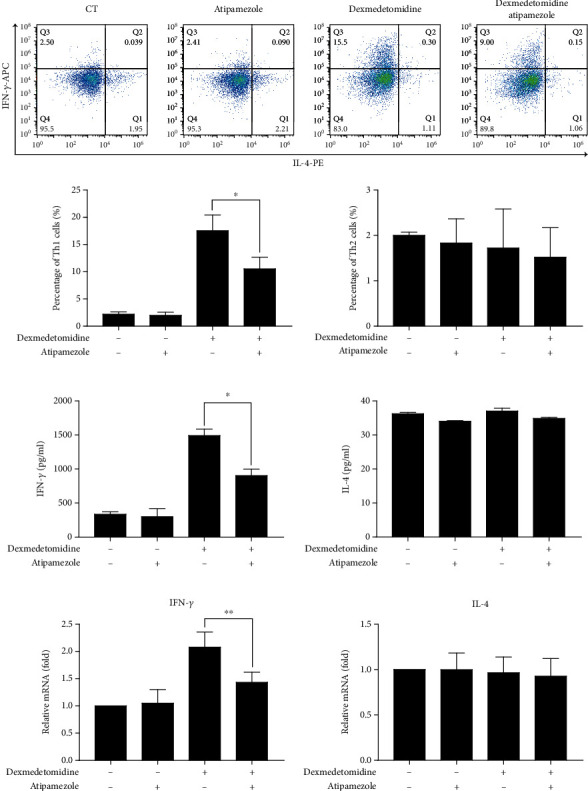
Atipamezole inhibits dexmedetomidine-induced Th1 cell differentiation and cytokine production. After being stimulated by anti-CD3/CD28 and rIL-2 for 3 days, CD4+ T cells were cultured in the presence or absence of dexmedetomidine (0.1 nM) and/or atipamezole (1 nM) for 24 hours. Th1 and Th2 cell concentrations (a) were measured by flow cytometry. The production (b) and expression (c) of related cytokines were detected by ELISA and real-time PCR, respectively. The data are representative of at least three independent experiments with consistent results. Student's *t*-test was performed to detect between-group differences. The results shown are the mean ± S.D. ^∗^*p* < 0.05, ^∗∗^*p* < 0.01.

T cells express both *α*1 receptor and *α*2 receptor, but cell proliferation and cytokine production are modulated by the *α*2 receptor [24]. Dexmedetomidine is a selective *α*2 receptor agonist and may function through the *α*2 receptor on the surface of T cells. To explore the possible function of the *α*2 receptor in Th1 cell differentiation, an *α*2 receptor antagonist atipamezole (1 nM) in combination with dexmedetomidine (0.1 nM) was added to irritate the cells. Pretreatment with atipamezole significantly attenuated the dexmedetomidine-induced content of Th1 cells (Figure 4(a)). The same phenomenon was observed in the concentration and expression of IFN-*γ* (Figure 4(b)). However, no matter whether atipamezole was used alone or in combination, there was no significant effect on Th2 cell differentiation or expression of IFN-*γ* (Figures 4(a) and 4(b)). These findings indicated that dexmedetomidine-induced Th1 cell differentiation required the participation of *α*2 receptors.

### 3.5. Atipamezole Prevents Dexmedetomidine-Induced STAT1 Phosphorylation and T-Bet Expression

**Figure 5:**
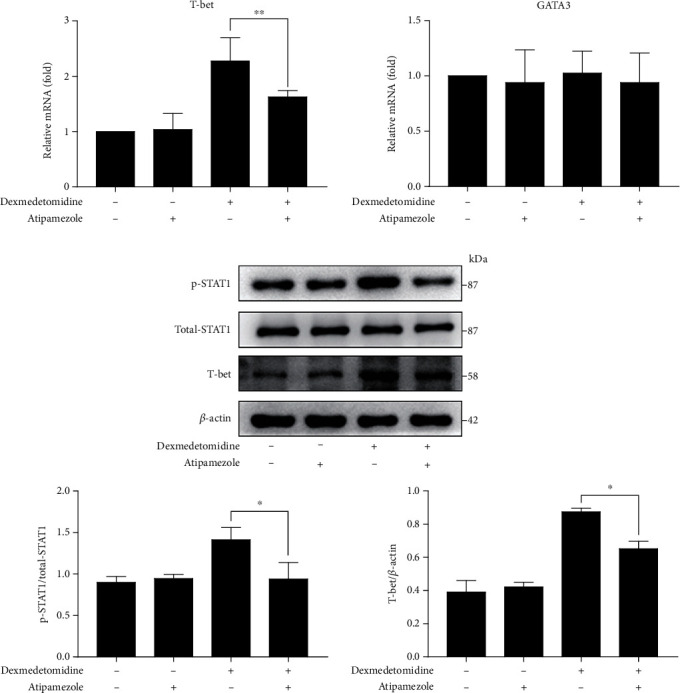
Atipamezole inhibits dexmedetomidine-induced STAT1 phosphorylation and T-bet expression. After stimulation by anti-CD3/CD28 and rIL-2 for 3 days, CD4+ T cells were cultured in the presence or absence of dexmedetomidine (0.1 nM) and/or atipamezole (1 nM) for 24 hours. T-bet and GATA3 (a) mRNA expressions were determined by real-time PCR. STAT1 phosphorylation and T-bet expression (b) were defined using Western blot. The data are representative of at least three independent experiments with consistent results. Student's *t*-test was performed to detect between-group differences. The results shown are the mean ± S.D. ^∗^*p* < 0.05, ^∗∗^*p* < 0.01.

Whether inhibition of the *α*2 receptor reverses the enhanced phosphorylation level of STAT1 or T-bet expression is unclear. According to the Methods and Material section, STAT1 phosphorylation and T-bet expression were measured after activated CD4+ T cells were stimulated by atipamezole. western blots proved that phosphorylation of STAT1 and expression of T-bet in cells treated with atipamezole and dexmedetomidine were significantly lower than in dexmedetomidine-stimulated CD4+ T cells (Figure 5(a)). The expression of T-bet at the mRNA level also followed this trend (Figure 5(b)). Regardless of the use of atipamezole alone or in combination, there was no significant change in GATA3 expression, suggesting that activation of the *α*2 receptor may not regulate Th2 cell differentiation (Figure 5(a)). These findings determined that dexmedetomidine induced the phosphorylation of STAT1 and T-bet expression via an *α*2 receptor-dependent mechanism.

### 3.6. Fludarabine Inhibits Dexmedetomidine-Induced Th1 Cell Differentiation and Cytokine Production

**Figure 6:**
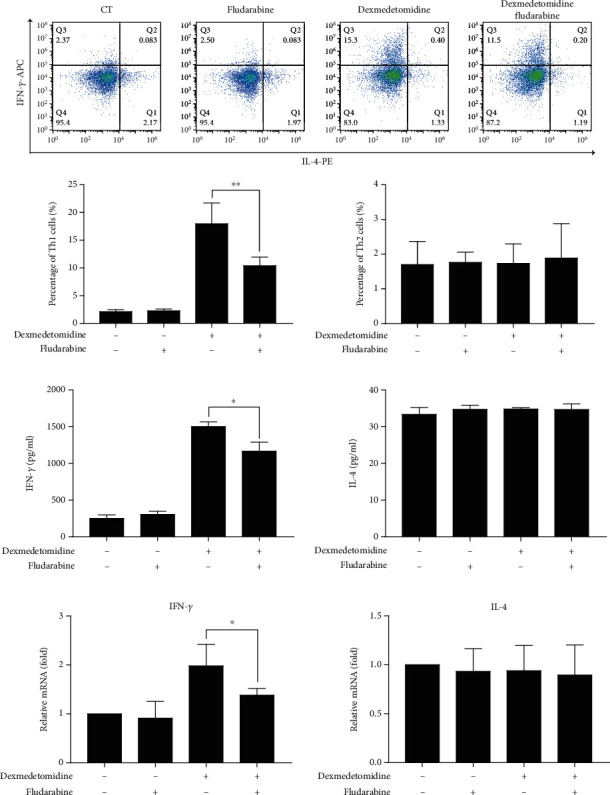
Anti-STAT1 (fludarabine) reverses dexmedetomidine-induced Th1 cell differentiation and cytokine production. After stimulation by anti-CD3/CD28 and rIL-2 for 3 days, CD4+ T cells were cultured in the presence or absence of dexmedetomidine (0.1 nM) and/or fludarabine (100 nM) for 24 hours. Th1 and Th2 cell concentrations (a) were measured by flow cytometry. The production (b) and expression (c) of related cytokines were detected by ELISA and real-time PCR, respectively. The data are representative of at least three independent experiments with consistent results. Student's *t*-test was performed to detect between-group differences. The results shown are the mean ± S.D. ^∗^*p* < 0.05, ^∗∗^*p* < 0.01.

Dexmedetomidine was shown to enhance Th1 cell differentiation and cytokine production by phosphorylating STAT1. We investigated whether the induction of dexmedetomidine in CD4+ T cells was STAT1 dependent. In this study, CD4+ T cells were cultured with fludarabine (100 nM) for 24 hours before dexmedetomidine (0.1 nM) was administered. Flow cytometry analysis revealed that dexmedetomidine-induced Th1 cell polarization was significantly inhibited by fludarabine (Figure 6(a)). In addition, inhibition of STAT1 phosphorylation by fludarabine reversed dexmedetomidine-induced IFN-*γ* secretion and expression (Figures 6(b) and 6(c)). These results demonstrated that dexmedetomidine-induced Th1 cell phosphorylation was dependent on phosphorylated STAT1.

### 3.7. Fludarabine Reduces T-Bet Expression in CD4+ T Cells by Inhibiting STAT1 Phosphorylation

**Figure 7:**
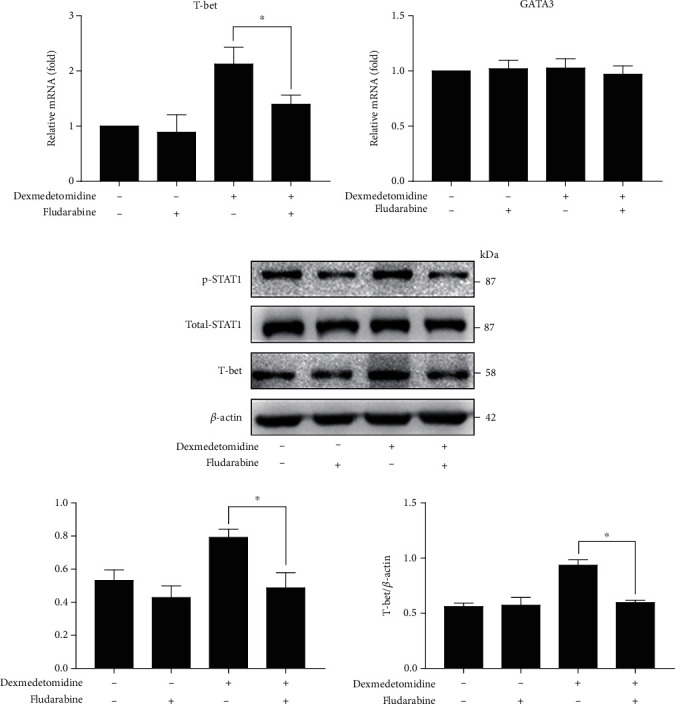
Anti-STAT1 (fludarabine) reverses dexmedetomidine-induced STAT1 phosphorylation and T-bet expression. After stimulation by anti-CD3/CD28 and rIL-2 for 3 days, CD4+ T cells were cultured in the presence or absence of dexmedetomidine (0.1 nM) and/or fludarabine (100 nM) for 24 hours. T-bet and GATA3 (a) mRNA expressions were determined by real-time PCR. STAT1 phosphorylation and T-bet expression (b) were defined using western blots. The data are representative of at least three independent experiments with consistent results. Student's *t*-test was performed to detect between-group differences. The results shown are the mean ± S.D. ^∗^*p* < 0.05, ^∗∗^*p* < 0.01.

Given that previous studies [19, 21] noted the contribution of STAT1 to Th1 cells, the effects of fludarabine on dexmedetomidine-induced STAT1 phosphorylation and T-bet expression were measured. To investigate whether fludarabine could reduce STAT1 phosphorylation and T-bet expression in dexmedetomidine-indicated CD4+ T cells, we treated cells with dexmedetomidine (0.1 nM) for 24 h with and without fludarabine. The results showed that fludarabine inhibited STAT1 phosphorylation and T-bet expression (Figures 7(a) and 7(b)), implicating that dexmedetomidine-induced T-bet expression could be reversed by inhibiting STAT1 phosphorylation.

## 4. Discussion

After the findings of this study, these phenomena directly suggested that dexmedetomidine induced the differentiation of Th1 cells in purified CD4+ T cells. We demonstrated that dexmedetomidine treatment activated STAT1 and induced T-bet expression *in vitro*. The *α*2 receptor inhibitor or STAT1 inhibitor reversed these phenomena, proving that dexmedetomidine-induced Th1 cell differentiation was dependent on the STAT1–T-bet pathway.

A few clinical studies [6, 7, 25] have claimed that dexmedetomidine increases the expression of INF-*γ* or the ratio of INF-*γ*/IL-4 in surgery patients. These studies indirectly indicated that dexmedetomidine could induce Th1 cell differentiation. Although INF-*γ* and IL-4 are considered characteristic cytokines of Th1 and Th2 cells [26], the mysterious microenvironment determines the uniqueness of cytokine secretion, such as natural killer cells producing INF-*γ* and activated mast cells secreting IL-4. To reduce interference from the complex secretion network, we cultured T cells with dexmedetomidine *in vitro*. This was beneficial to directly prove that dexmedetomidine could induce Th1 cell differentiation and enhance its function.

Dexmedetomidine is a selective *α*_2_ adrenergic receptor agonist [27]. Due to the widespread existence of *α*2 receptors in the cardiovascular system and nervous system, it has been used in the clinic mainly for sedation, anesthesia, and analgesia [28]. Evidence [29] has supported that dexmedetomidine regulates immune cells, but the underlying mechanism in Th1 cell differentiation is unclear. One possibility is that dexmedetomidine inhibits the secretion of norepinephrine by activating the central nervous system *α*2 adrenergic receptors and then indirectly regulates Th1 cell differentiation [30]. However, it does not explain whether dexmedetomidine directly stimulates the *α*2 receptors on T cells [24]. Our study proved that dexmedetomidine induced Th1 differentiation by directly stimulating the *α*2 receptor on the surface of T cells because the *in vitro* coculture of *α*2 receptor inhibitors with cells reversed the effect of dexmedetomidine. Furthermore, a published study [31] indicated that activation of *α*2 adrenergic receptors regulates ERK expression and upregulates p38 MAPK activation. The activated p38 MAPK increases the phosphorylation of STAT1 through the stress activation pathway [32] and maybe the regulatory mechanism of dexmedetomidine. In contrast, administration of atiprazole inhibits STAT1 phosphorylation induced by dexmedetomidine proved that STAT1 phosphorylation was a downstream event after activation of *α*2 adrenergic receptors. STAT protein participates in regulating physiological processes such as cell proliferation, differentiation, apoptosis, and angiogenesis [33]. STAT1, a member of the STAT family, has SH2 and SH3 domains containing phosphorylation sites on tyrosine residues. Phosphorylation occurs after activation of STAT1, promoting the transport of STAT1 from the cytoplasm to the nucleus [34]. Phosphorylated STAT1 can reside in the nucleus to regulate the transcription process and differentiation of T cells [35]. This is consistent with our experimental phenomenon because dexmedetomidine-induced STAT1 phosphorylation and Th1 differentiation were reversed by STAT1 inhibitor administration. The inhibition of Th1 differentiation in STAT1^−/−^ mice is attributed to decreased expression of T-bet [36], indicating that STAT1 may regulate T-bet expression. T-bet, as one of the transcription factors, is essential for Th1 cell-characteristic gene expression [37] to promote Th1 cell differentiation [17]. In our study, it was found that the phosphorylation level of STAT1 and the expression of T-bet were increased in T cells stimulated by dexmedetomidine because T-bet expression enhanced by phosphorylated STAT1 could induce the differentiation of Th1 cells. Furthermore, our study also found that STAT1 regulated the expression of IFN-*γ*, especially inhibiting the activity of STAT1. Activated STAT1 directly participates in the IFN-*γ* signaling pathway [38, 39], inhibiting IL-2 and IFN-*γ* synergistically and promoting the development of Th1 cells. Interestingly, dexmedetomidine neither enhanced nor inhibited GATA3 expression and Th2 cell differentiation, similar to changes in Th2 cells in STAT1^−/−^ mice [36]. One possibility is that STAT1 is not involved in regulating GATA3 expression and is related to stimulation under neutral conditions in vitro. Dexmedetomidine induces Th1 cell differentiation in a dose-dependent manner, but there is an optimal concentration. High concentrations of *α*2 receptor agonists could activate the Fas/Fasl pathway to induce a small amount of apoptosis in spleen cells [40]. The detailed mechanism by which *α*2 receptors regulate STAT1 needs further exploration, but it is clear that *α*2 receptors regulate the phosphorylation of STAT1.

## 5. Conclusion

In summary, our study demonstrated that the STAT1–T-bet signaling pathway activated by *α*2 receptors is a potential mechanism in dexmedetomidine-induced Th1 cell differentiation. The effect of dexmedetomidine in influencing CD4+ T cells toward a Th1 cell fate suggested a potential connection between the nervous system and the immune system.

## Data Availability

The data used to support the findings of this study are included within the article.
